# Open reduction and internal fixation of modified Mason type III/IV radial head fractures with more than three fragments: an analysis of the clinical outcome and reoperation rate

**DOI:** 10.1007/s00590-023-03772-y

**Published:** 2023-11-14

**Authors:** Timothée Helmstetter, Mauro Maniglio, Paolo Fornaciari, Moritz Tannast, Philippe Vial

**Affiliations:** 1 University of Fribourg Medical School, Chemin du Musée, 1700 Fribourg, Switzerland; 2 Department of Orthopedics and Traumatology, Fribourg Cantonal Hospital, Chemin des Pensionnats 2-6, 1752 Villars-sur-Glâne, Switzerland

**Keywords:** Elbow, Radial head, Mason III/IV, Osteosynthesis, ORIF, Fracture

## Abstract

**Purpose:**

It is generally accepted that a radial head fracture (RHF) with more than three parts is not suitable for repair; therefore, most authors suggest straightforward radial head arthroplasty (RHA). With up to 20% risk for reoperation after RHA, improvement in reduction and fixation techniques may represent a valuable alternative before further extending the indications for arthroplasty. To determine the functional results and radiological failure rate after osteosynthesis of multi-fragmentary RHF with more than three articular fragments. We specifically determined (1) the one-year Broberg and Morrey functional elbow score, (2) duration of fracture healing, (3) complication rate, and (4) number of patients converted to RHA.

**Methods:**

This study is a retrospective single-center case series. All patients who underwent primary osteosynthesis for RHF between 2012 and 2019 were included. Nine patients with an average age of 52 years had an average clinical and/or radiological follow-up of 49 months.

**Results:**

The preoperative imaging identified nine fractures with four fragments. Three patients underwent osteosynthesis with plates and screws, whereas six patients underwent osteosynthesis with only screws. The mean Broberg and Morrey score was 95 points. Overall, eight of the nine patients had satisfactory results. All patients retained their radial heads and showed radiological fracture healing. Only two patients presented with low-grade complications requiring no further surgery.

**Conclusion:**

Our study showed that osteosynthesis of RHF with up to four fragments can achieve good functional results with a low complication rate and seems to be a valid alternative to RHA.

## Introduction

Radial head fractures are the most common type of elbow fractures. They represent approximately 75% of all proximal forearm fractures and 2%–5% of all adult fractures [1].

The treatment of type III/IV comminuted radial head fractures according to Mason Johnston’s classification remains controversial. It is generally accepted that radial head fractures with more than three parts are unsuitable for repair. According to the literature published by Ring et al. [2, 3], 92% of patients with a radial head fracture with more than three articular fragments had an unsatisfactory result after open reduction and internal fixation (ORIF); therefore, most authors suggest a straightforward radial head arthroplasty (RHA) [3–6].

However, the management of multi-fragmentary fractures of the radial head with radial head replacement seems to have a 20% risk for reoperation due to stiffness and painful loosening [7].

Facing this high complication rate, improvement in reduction and fixation techniques may represent a valuable alternative before further extending the indications for arthroplasty. The purpose of this retrospective study was to determine the functional results and radiological failure rate following ORIF of displaced fractures of the radial head types III and IV according to Mason/Johnston classification using headless compression screws (HCS) and/or low-profile 1.2–2.3 locking compression plates. We specifically determined (1) the minimum one-year Broberg and Morrey functional elbow score, (2) duration of fracture healing, (3) complication rate, and (4) number of patients who converted to RHA.

This study was approved by the local Institutional Review Board (CER-VD Project-ID 2020-00488).

## Materials and methods

### Study design and patient selection

The present study was a clinical and radiographic follow-up study of a consecutive case series (level of evidence IV) of patients who had undergone primary ORIF with headless and/or countered plates and screws for a radial head fracture. Of 114 consecutive patients diagnosed with acute radial head fractures at our institution between January 2012 and June 2019, we included 35 skeletally mature patients with Mason-III/IV fractures.

**Fig. 1:**
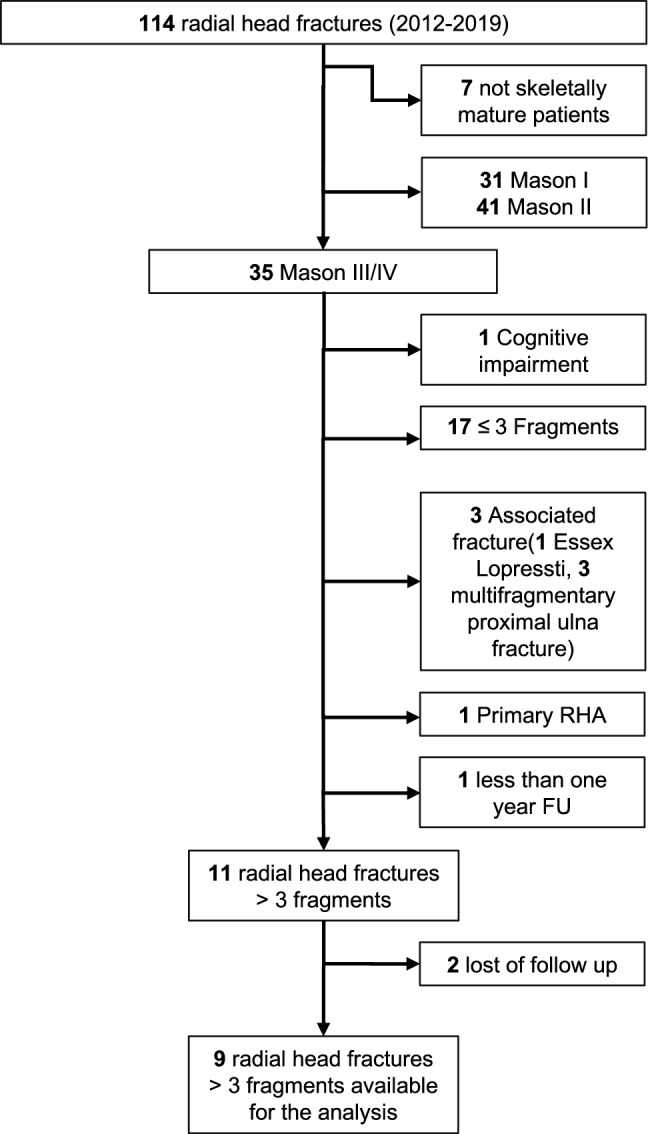
Chart flow of radial head fracture between 2012 and 2019

We excluded all patients with severe cognitive impairment (*n* = 1), radial head fracture with three or less intra-articular fragments (*n* = 17), concomitant upper limb fractures (*n* = 4 of which one with associated Essex-Lopresti and three with associated multi-fragmentary proximal ulna fracture on the same side), primary prosthesis implantation (*n* = 1), less than 1-year follow-up (*n* = 1), leaving 11 patients. Of these, one refused to participate and one moved to a foreign country and was lost to follow-up, leaving nine elbows for evaluation (Fig. 1). The patients were considered unreachable if no response was obtained after three consecutive phone calls on different days of the week and no further contact information from alternative sources was available. Informed consent was obtained from all individual participants included in the study.

### Demographics

The cohort comprised five men and four women with a mean age of 50 (range, 28–71) years. Six patients were right-handed, and half of them injured the dominant side. A summary of the patient demographics is presented in Table 1.

**Table 1:** Patient’s demographics, fracture characteristics, intra-operative parameters, osteosynthesis modalities

Parameter	Case								
	1	2	3	4	5	6	7	8	9
*Demographic*									
Age (years)	35	47	63	38	47	71	60	58	28
Sex	M	M	F	M	M	F	F	F	M
Side	Left	Right	Left	Right	Right	Right	Left	Right	Right
Dominant side	Yes	Yes	Yes	–	–	Yes	–	–	Yes
Profession	Jurist	Optician	Secretary	Works supervisor	Electrician	Pensioner	Pensioner	Office employee	Farmer employee
*Fracture characteristics*									
Mason type	III	III	III	IV	III	III	IV	IV	IV
Number of fragments	4	4	4	4	4	4	4	4	4
Associated neck fracture	Yes	–	–	–	Yes	–	–	Yes	–
Coronoid fracture type (Regan–Morrey)	–	–	II	II	II	I	II	I	I
Associated ligament injuries	–	–	–	LCL/MCL	LCL/MCL	LCL	LUCL	LUCL	LCL
*Intraoperative parameters*									
Time to surgery (days)	3	15	5	8	7	11	9	4	8
Surgery duration (min)	93	63	80	125	165	110	115	120	150
*Osteosynthesis modality*									
Headless compression screw (HCS) only/number of screws	No/1	Yes/4	Yes/4	Yes/3	No/3	Yes/5	Yes/4	No/4	Yes/8
Plate osteosynthesis	Straight and Y-plate	–	–	–	T-plate	–	–	Y-Plate	–
*Coronoid and ligament repair*									
Coronoids anchor fixation	–	–	Yes	Yes	Yes	–	–	Yes	–
Ligament repair	–	–	–	LCL/MCL	LCL/MCL	LCL	LUCL	LUCL	LCL

*LCL* lateral collateral ligament, *MCL* medial collateral ligament, *LUCL* lateral ulnar collateral ligament

### Patient management

The initial diagnosis in the emergency department was made via radiographic examination. In the case of initial elbow dislocation, reduction was performed in the emergency ward under sedation, followed by radiological evaluation. All patients received a temporary posterior elbow splint.

### Fracture and ligament lesion characteristics

The fractures were classified according to the Mason classification modified by Johnston [8]. Five fractures were Mason-III, and four were Mason-IV. Preoperative computed tomography (CT) revealed nine fractures with four fragments. Three patients also had associated neck fracture. According to the Regan and Morrey classification, coronoid fractures were present in seven patients comprising three type-I and four type-II fractures. Associated ligament injuries are summarized in Table 1 and were present in six patients.

Surgery was indicated for comminuted displaced radial head fractures with mechanical block-to-motion.

### Surgical technique

The patient was placed in the supine position on the operating table. The affected limb was placed on the chest or table with the forearm in pronation. The lateral epicondyle and the radial head were palpated. An incision was made from the posterior aspect of the lateral epicondyle, distal to the posterior border of the ulna, approximately 5–6 cm from the tip of the olecranon.

A slightly ventrally shifted approach was preferable with respect to the radio-humeral joint; we used an approach between the classic Kocher and Kaplan's approach [9] with a splitting of the extensor digitorum communis.

With the correct height of the muscle section, it was possible to avoid a longer incision (≈ 2 cm in all cases), reducing complications such as stiffness. In addition, the incision should not be extended distally to the neck of the radius to avoid damage to the radial nerve because it extends anterolaterally along the joint capsule.

Particular attention was paid to avoid a complete section of the annular ligament if still intact. The partial preservation of the ligament can help to achieve and maintain the fragment reduction. Once on the fractured fragments, the main part of the procedure is dedicated to anatomical fragments reduction. Reduction was performed with a dentist hook and was considered anatomical if a step or space < 1 mm was present on inspection and palpation. Gap reduction is usually less demanding than step-dislocation reduction. Impaction of the fragments was reported in all treated fractures. Impaction may only be present in the main fragment or involve small adjacent fragments. Temporary stabilization with K-wires is not mandatory and can sometimes critically reduce the field of freedom in pro-supination required for fixation, or worse, still occupy important cartilage surfaces of the head where headless cortical screws could be placed.

**Fig. 2:**
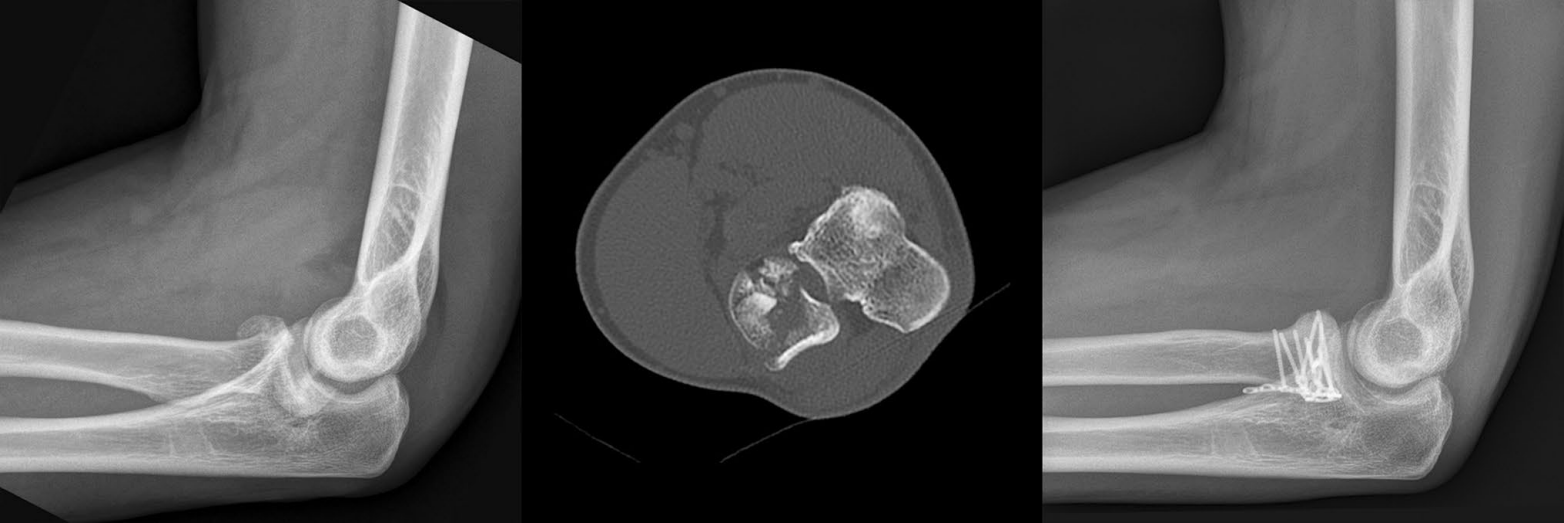
Preoperative radiograph, axial CT scan and postoperative radiograph of a 4-part radial head fracture treated by ORIF with plates

Thus, correct positioning of the HCS was decisive. We used 1.5 mm HCS Synthes (DePuy Synthes® Raynham, MA, USA). When drilling and tightening screws in a plane parallel to the surface of the radial head, care must be taken to not lose the reduction, which would easily create gaps between the fragments. The tightening of the screws must be sufficient to create a stable construction; however, shear forces must be avoided. Insertion of screws perpendicular to the fracture line should be targeted whenever possible. After reducing and securing the joint surface, head–neck fixation was initiated. Creating a stable structure between the head and neck is important to withstand axial forces. In the case of neck fractures, we obtained it with a small T-plate or Y-plate Aptus® Trilock 1.2/1.5, and 2.0/2.3 systems (Medartis, Basel, Switzerland) (Fig. 2). When necessary, additional HCS screws can be placed under the plate. Alternatively, axial stability could be achieved without plating, by using direct HCS through the angle of the articular surface of the radial head and aiming at the cortical neck bone or immediately distal to the neck. The thread of the screw tip anchors in the contralateral cortical bone, whereas the thread of the head in the proximal cortical bone creates some compression at the radial head creating some. Strengthening this system with two or three screws provides sufficient axial stability, similar to a table with three crossed legs (“Tripod technique”).

Regan and Morrey type-II coronoid fractures were addressed before radial head fractures. We fixed the coronoid processes with the anchor Healicoil 4.5 (Smith&Nephew®, London, England). The insertion of the anchor through a lateral approach is done by positioning the impacted part of the radial head in front of the coronoid and bending the elbow slightly more than 90 degrees. The Regan and Morrey type-I fractures were treated conservatively.

Collateral ligament ruptures were addressed as necessary. The lateral ulnar collateral ligament (LUCL) and lateral collateral ligament complex were fixed with Healicoil 4.5 anchors (Smith & Nephew®, London, England) and Krackow sutures. The anterior medial collateral ligament (aMCL) stability was assessed intraoperatively using the valgus stress test. Owing to instability, the aMCL was fixed with a transosseous suture through the medial epicondyle or anchors.

### Intraoperative parameters and material

The mean duration until surgery was 8 (range 3–15) days, while the mean duration of the surgical procedure was 113 (range 63–165) minutes. Three patients underwent osteosynthesis with plate and screws and six patients with HCS only (Table 1). Additional surgical procedures, such as coronoid fixation and ligament repair, are summarized in Table 1.

### Postoperative protocol

During the 6 post-operative weeks, only limited flexion/extension of 90-0-0 and free pro-supination was permitted. All patients underwent physiotherapy for early mobilization. We applied postoperatively a posterior elbow splint at 90° elbow flexion for 3 weeks. The patients had to remove the splint at home during the day in a safe environment and were allowed to move the elbow with respect of the prescribed limitation. However, they had to wear it at night and leave home. No charges were allowed until radiological consolidation was achieved.

### Postoperative clinical and radiological follow-up

Patients were followed up at 6 weeks and 3, 6, and 12 months clinically, with a mean clinical follow-up of 49 (range 12–87) months after ORIF. Radiological follow-up consisting of biplanar radiographs was performed until at least the fracture healed.

Clinical evaluation was based on the Broberg and Morrey elbow functional scores at the last follow-up. Flexion–extension was measured in neutral position and pro-supination with 90° elbow flexion. During the pro-supination examination, the patient held a metal bar in his hand, and the angle between the bar and the horizontal plane was measured. Elbow instability was tested using the valgus and varus stress tests at each follow-up and compared with the healthy side. The clinical results were grouped as "excellent,” "good,” "fair," and "poor,” corresponding to a score of 100–95, 94–80, 79–60, and < 60, respectively. The results classified as “fair” and “poor,” as well as a forearm rotation < 100° were considered unsatisfactory.

Radiologically, antero-posterior and lateral views of the elbow were obtained in the neutral forearm position and compared with postoperative radiographs. Fixation failure was defined in case of ≥ 1-mm displacement of the fragments. Non-union was defined as the failure of radiographic consolidation on radiographs after six months. Fixation failure and non-union were considered unsatisfactory. The complications were graded according to the method described by Sink et al. [10].

## Results

*(1) Broberg and Morrey functional elbow score and clinical analysis*

The mean Broberg and Morrey scores were 95 (range 75–100) points at a mean clinical follow-up of 49 (range 12–87) months after ORIF. The results were rated excellent in six patients, good in two, and fair in one at the final follow-up. Overall, eight of the nine patients had satisfactory results (Table 2).

**Table 2:** Clinical outcomes

Case	Flexion/extension (°)	Pro/supination (°)	Broberg and Morrey score
1	150/0/10	90/0/90	100
2	135/35/0	85/0/85	93
3	135/5/0	85/0/90	100
4	143/9/0	55/0/88	91
5	110/25/0	75/0/60	75
6	135/0/2	90/0/90	99
7	135/9/0	90/0/90	98
8	135/6/0	84/0/90	99
9	140/5/0	90/0/90	100

The mean arc of elbow flexion–extension motion was 125° (range 85°–160°), with a mean flexion of 135° (range 110°–150°) and a mean extension deficit of 10° (range 0°–35°). The mean arc of forearm pro-supination was 169° (range 135°–180°), with a mean pronation of 83° (range 55°–90°) and a mean supination of 86° (range 60°–90°). All the elbows were stable.

*(2) Duration of fracture healing*

All patients showed radiological healing of the fractures at a mean of 9 (range, 6–13) weeks.

*(3) Complications rate*

According to Sink et al. [10] complication classification system for orthopedics surgery, two of the nine patients developed complications, one class I and one class II.

Case 5 showed anterior heterotopic ossification (HO) at the 6-week radiological follow-up, with functional limitation in both planes of motion graded as grade-IIC according to the Hastings classification (flexion/extension 100-70-0 and pronation/supination 40-0-30). After the administration of indomethacin for one month, HO remained stable. The patient’s motion improved with intensive physiotherapy and was asymptomatic (flexion/extension 110-25-0 and pronation/supination 75-0-60 at 1-year follow-up).

Case 6 showed displacement of a fragment of the radial head on postoperative radiography. An additional CT scan showed an acceptable construction, and no additional surgeries were performed. During three weeks, the patient was immobilized with an elbow cast in a full supination cast. No further displacement was noted on follow-up radiography, and Broberg and Morrey scores were rated as excellent at the final follow-up (Table 2).

No cases of infection were registered. No implant removal was necessary.

*(4)* Number of patients converted to RHA

No patients needed conversion to RHA.

## Discussion

To the best of our knowledge, this is the first study on the functional outcomes after ORIF of radial head fractures, focusing specifically on fractures with more than three fragments.

Multi-fragmentary Mason-III/IV radial head fractures remain a surgical challenge. According to the literature, such fractures should not be treated by radial head resection but either by reconstruction or replacement because of persistent elbow pain, instability, and loss of strength [11, 12, 22]. Based on our experience, we prefer to perform primary ORIF after radial head fractures with more than three fragments. In our case series, all patients were treated with plating and/or headless screws. Eight of the nine patients had satisfactory results, with a mean Broberg and Morrey functional score of 95 points and few complications. The mean range of motion was 135° of flexion with an extension deficit of 10° and pronation/supination of 83°-0-86°. All fractures healed and no conversion to RHA was necessary at the mean clinical follow-up of 49 months.

Comparing the results of our cohort with those of previous studies, we found similar [13–16] or better [2, 5] mean Broberg and Morrey scores at final follow-up. After overviewing the literature, we found only a few studies in which the number of fragments were counted [2, 15–17]. By comparing groups of radial head fractures with more than three fragments, we found similar results to those of other studies [15–17], except for those by Ring et al., Chen et al., and Ruan et al. [2, 4, 5], who found a lower satisfaction rate of 1 of 14, 15 of 23, and 1 of 8, respectively. This difference can be explained by analyzing the inclusion criteria. In their cohort, Ring et al. had indeed 48% of patients, with more complex trauma than an isolated radial head fracture, such as concomitant Essex-Lopresti lesions or Monteggia fractures that can lead to bad functional results due to stiffness [18]. Patients with complex injuries were excluded to avoid potential bias.

Regarding the range of motion, we had similar results concerning flexion and extension deficits, but the mean pro-supination seemed to be better (Table 3). We hypothesize that the minimally invasive approach, which includes fewer soft tissue dissections, the extensor split interval that preserves the lateral ulnar collateral ligament, and respect for the integrity of the annular ligament during surgery, could explain the reduced postoperative stiffness and instability. Only case 5 in our cohort showed unsatisfactory results. This fair Broberg and Morrey score was due to diminished pro-supination caused by HO, a complication that leads to joint stiffness and elbow disability. However, with a flexion/extension of 110°/25°/0 and a pro-supination of 75°/0/60°, it was sufficient to perform his regular professional activity as an electrical technician.

**Table 3:** Literature overview of osteosynthesis treatment for similar multifragmentary radial head fractures cohort

References	Number of cases	Type of fracture (number of cases)	Number of fragments (number of cases)	ORIF technique (number of cases)	Mean Follow-up (months)	Broberg and Morrey score	Satisfaction rate	Range of motion F/Ext deficit in degree Pro/sup in degree	Complications	Reoperation rates	Conversion rate in RHA
Chen [13]	34	Mason III (25) Mason IV (9)	NK	Screws, absorbable rods, low-profile T locking plate	12	86.50	22/34	NK	Ankylosis 4/34 HO 1/34	0	0
Crönlein [28]	20	Mason III (14) Mason IV (6)	NK	Low-profile locking plate (Aptus elbow 2.0)	30 (18–53)	NK	20/20	NK	0	0	0
Burkhart [17]	6	Mason III (5) Mason IV (1)	4 fragments (4) 5 fragments (2)	Medartis rim plates: 3 Medartis Buttress plates: 3	15 (5–21)	NK	5/6	4 fragments: 130°/12° 66°/68° 5 fragments: 137°/12° 71°/63°	1/6 HO	1/6 revision for radial head excision	0
Chen [5]	23	Mason III	NK	18 screws, 5 AO T mini-plates	34 (12–60)	72.4	15/23	NK	11/23	4/23	4/23
Businger [14]	6	Mason III (2) Mason IV (4)	NK	Low-profile T locking plate and screws	117 (86–144)	97 (93–100)	6/6	141°/6° 79°/70°	6/6 annular ligament irritation	6/6 removal	0
Ruan [4]	8	Mason III	NK	Cannulated screws and K wires	14 (10–21)	NK	1/8	NK	4/8 non-union	4/8 revision for radial head excision	0
Ozkan [29]	12	Mason III (8) Mason IV (4)	NK	Mason III: 1 K-wires, 6 cannulated, 1 miniplate Mason IV: 1 K-wires, 1 cannulated, 2 miniplate low profile	Mason III: 53 (42–72) Mason IV: 51 (42–65)	NK	Mason III 7/8 Mason IV 2/4	Mason III: 143°/5.6° 75°/88° Mason IV: 111°/18.6° 60°/68°	Mason III: 1 non-union Mason IV: 1 implant failure	2/12 Mason II: 1/8 nonunion Mason IV: 1/4 excision and RHA	Mason III: 1/8 Mason IV 1/4
Nalbantoglu [15]	25	Mason III/IV	≤ 3 fragments (20) > 3 fragments (5)	19 low-profile T plate and screws, 1 headless screw 5 radial head plate	26 (12–40)	87.2 (75–100)	≤ 3 fragments 15/20 > 3 fragments 5/5	136°/4.9° 63°/75°	1 non-union 3HO 1 painful material	1/6 painful material	0
Ikeda [16]	13	Mason III/IV	≤ 3 fragments (7) > 3 fragments (6)	8 low-profile 3 Herbert screw 2 combination	36 (24–48)	90.7 (73–100)	12/13 patients	134°/7° 73/85	0	9 plate removal	0
Ring [2]	26	Mason III/IV	≤ 3 fragments (12) > 3 fragments (14)	22 plates 4 screws	44 (25–102)	86 (51–99)	≤ 3 fragments 11/12 > 3 fragments 1/14	129°/18° 67°/59°	3 material loosening 7 non-union	10/26 revision for excision (9 of 14 > 3 fragments)	0
Esser [30]	15	Mason III (9) Mason IV (6)	NK	AO mini-screws, mini-AO plates, Herbert’s screws	87.6 (12–168)	Mason III: 97 Mason IV:83	Mason III 9/9 Mason IV 4/6	Mason III: 138°/3° Mason IV: 132°/13° 85°/70°	2 non-union	Mason IV 2/6 revision for excision	0

With a low complication rate, no pseudarthrosis, and no conversion to RHA or radial head excision, our secondary outcomes were comparable to or better than those of previous reports on osteosynthesis of comminuted radial head fractures (Table 3).

Furthermore, in contrast to other studies, we did not remove any materials. The causes of removal are inhomogeneous, such as annular ligament irritation, painful hardware, or the patient’s wish [14, 16], but we hypothesize that our results are a consequence of the use of headless screws or low-profile plates that do not disturb pro-supination.

Since the publication of Ring et al. [2], many surgeons follow the recommendation that if more than three fragments are present, osteosynthesis should no longer be considered a therapeutic option, and primary RHA should be performed.

RHA following a severely comminuted fracture has shown inhomogeneous results in the current literature. Some authors have reported a high satisfaction rate with a low reoperation rate [13, 19–21], whereas others have reported a reoperation rate of up to 28% [22–24].

These results are supported by one recent meta-analyses of long-term results after RHA for radial head fractures, in which Davey et al. [7] showed reoperation rates of 20% (86 of 432 with 65 patients who underwent prosthesis removal) at 8 years of follow-up.

The most prevalent reasons for revision or failure of RHA are symptomatic loosening, stiffness, pain, oversizing or overlengthening, dissociation of the prosthesis, erosions of the capitellum, and progressive symptomatic osteoarthritis of the ulno-humeral joint [25]. The radial head shows many anatomical variations. Owing to its non-circular shape, there is currently no implant that matches the exact shape and reproduces the biomechanics of the radial head [26]. Hence, even when the prosthesis is perfectly implanted, the radial head and capitellum no longer exhibit an anatomical relationship.

Considering that the mean patient age at radial head replacement was 50 years, even a long-term moderate rate of prosthesis removal could significantly affect the quality of life of this active patient population [27]. These arguments favor preservation of the radial head. Therefore, we believe that paying attention to the details in the preparation of the approach and reduction could greatly and reproducibly improve the results after ORIF of multi-fragmentary radial head fractures.

Our study has several limitations. First, this was a non-randomized retrospective study with no comparative groups. Second, despite the long recruitment period, only a small number of patients were included. However, multi-fragmentary radial head fractures are uncommon, and those with more than three fragments are even rarer. Our cohort was composed only of fracture cases with four fragments. Third, the modality of osteosynthesis is not uniform, but corresponds well to a pragmatic, real-life condition in which surgeons must adapt the fixation material to each fracture.

## Conclusions

Our study shows that ORIF of radial head fractures with up to four fragments can achieve good functional results with a low complication rate and seems to be a valid alternative to radial head replacement. In our opinion, the presence of more than three fragments as a cut-off for the primary implantation of an RHA must be challenged.

## References

[1] Jackson JD, Steinmann SP (2007). Radial head fractures. Hand Clin.

[2] Ring D, Quintero J, Jupiter JB (2002). Open reduction and internal fixation of fractures of the radial head. J Bone Jt Surg Am.

[3] Ring D (2011). Radial head fracture: open reduction-internal fixation or prosthetic replacement. J Shoulder Elbow Surg.

[4] Ruan H-J, Fan C-Y, Liu J-J, Zeng B (2009). A comparative study of internal fixation and prosthesis replacement for radial head fractures of Mason type III. Int Orthop.

[5] Chen X, Wang S, Cao L, Yang G, Li M, Su J (2011). Comparison between radial head replacement and open reduction and internal fixation in clinical treatment of unstable, multi-fragmented radial head fractures. Int Orthop.

[6] Sun H, Duan J, Li F (2016). Comparison between radial head arthroplasty and open reduction and internal fixation in patients with radial head fractures (modified Mason type III and IV): a meta-analysis. Eur J Orthop Surg Traumatol.

[7] Davey MS, Davey MG, Hurley ET, Galbraith JG, Molony D, Mullett H (2021). Long-term outcomes of radial head arthroplasty for radial head fractures—a systematic review at minimum 8-year follow-up. J Shoulder Elbow Surg.

[8] Johnston GW (1962). A follow-up of one hundred cases of fracture of the head of the radius with a review of the literature. Ulster Med J.

[9] Han F, Teo AQA, Lim JC, Ruben M, Tan BHM, Kumar VP (2016). Outcomes using the extensor digitorum communis splitting approach for the treatment of radial head fractures. J Shoulder Elbow Surg.

[10] Sink EL, Leunig M, Zaltz I, Gilbert JC, Clohisy J (2012). Reliability of a complication classification system for orthopaedic surgery. Clin Orthop Relat Res.

[11] Zarifian A, Rahimi Shoorin H, Hallaj Moghaddam M, Fathi Vavsari M, Gharedaghi M, Moradi A (2018). The best option in treatment of modified mason type III radial head fractures: open reduction and internal fixation versus radial head excision. Arch Bone Jt Surg.

[12] Hall JA (2005). Posterolateral rotatory instability of the elbow following radial head resection. J Bone Jt Surg (Am).

[13] Chen H-W, Tian J-L, Zhang Y-Z (2021). Therapeutic effect of resection, prosthetic replacement and open reduction and internal fixation for the treatment of mason type III radial head fracture. J Investig Surg.

[14] Businger A, Ruedi TP, Sommer C (2010). On-table reconstruction of comminuted fractures of the radial head. Injury.

[15] Nalbantoglu U, Kocaoglu B, Gereli A, Aktas S, Guven O (2007). Open reduction and internal fixation of mason type iii radial head fractures with and without an associated elbow dislocation. J Hand Surg Am.

[16] Ikeda M, Sugiyama K, Kang C, Takagaki T, Oka Y (2006). Comminuted fractures of the radial head: comparison of resection and internal fixation. J Bone Jt Surg.

[17] Burkhart KJ, Gruszka D, Frohn S, Wegmann K, Rommens PM, Eicker CM (2015). Winkelstabile Plattenosteosynthese des Radiuskopfes. Unfallchirurg.

[18] Ring D (2013). Monteggia fractures. Orthop Clin N Am.

[19] Laun R, Tanner S, Grassmann J-P, Schneppendahl J, Wild M, Hakimi M (2019). Primary cemented bipolar radial head prostheses for acute elbow injuries with comminuted radial head fractures: mid-term results of 37 patients. Musculoskelet Surg.

[20] Sershon RA, Luchetti TJ, Cohen MS, Wysocki RW (2018). Radial head replacement with a bipolar system: an average 10-year follow-up. J Shoulder Elbow Surg.

[21] Marsh JP, Grewal R, Faber KJ, Drosdowech DS, Athwal GS, King GJW (2016). Radial head fractures treated with modular metallic radial head replacement. J Bone Jt Surg.

[22] Raven TF, Schönewald M, Doll J, Banken L, Schmidmaier G, Moghaddam A (2019). Evaluation of MoPyC-prosthesis implantation and the use of angular stable plates in the treatment of comminuted radial head fractures. J Orthop.

[23] Duckworth AD, Wickramasinghe NR, Clement ND, Court-Brown CM, McQueen MM (2014). Radial head replacement for acute complex fractures: what are the rate and risks factors for revision or removal?. Clin Orthop Relat Res.

[24] Nolte P-C, Tross A-K, Groetzner-Schmidt C, Jung MK, Porschke F, Grützner PA (2021). Risk factors for revision surgery following radial head arthroplasty without cement for unreconstructible radial head fractures. J Bone Jt Surg.

[25] Marcheix P-S, Cuenca C, Vergnenegre G, Mabit C, Hardy J, Charissoux J-L (2021). Factors influencing the mid-term radiological and functional outcomes of 41 post-fracture bipolar radial head arthroplasty cases at a mean follow-up of 87 months. Orthop Traumatol Surg Res.

[26] van Riet RP, Van Glabbeek F, Neale PG, Bortier H, An K-N, O’Driscoll SW (2003). The noncircular shape of the radial head. J Hand Surg Am.

[27] Swensen SJ, Tyagi V, Uquillas C, Shakked RJ, Yoon RS, Liporace FA (2019). Maximizing outcomes in the treatment of radial head fractures. J Orthop Traumatol.

[28] Crönlein M, Zyskowski M, Beirer M, Imhoff FB, Pförringer D, Sandmann GH (2017). Using an anatomically preshaped low-profile locking plate system leads to reliable results in comminuted radial head fractures. Arch Orthop Trauma Surg.

[29] Ozkan Y, Oztürk A, Ozdemir RM, Aykut S, Yalçin N (2009). Open reduction and internal fixation of radial head fractures. Ulus Travma Acil Cerrahi Derg.

[30] Esser RD, Davis S, Taavao T (1995). Fractures of the radial head treated by internal fixation: late results in 26 cases. J Orthop Trauma.

